# Short-Term Outcomes in Patients With Coexistence of COVID-19 Infection and Vitamin D Deficiency: A Large Cohort Study

**DOI:** 10.7759/cureus.71952

**Published:** 2024-10-20

**Authors:** Yi-Ju Chan, Chia-Chen Chen, Jheng-Yan Wu

**Affiliations:** 1 Surgery, Department of Surgery, Division of Plastic and Reconstructive Surgery, Chi Mei Medical Center, Tainan, TWN; 2 Endocrinology and Metabolism, Department of Internal Medicine, Chi Mei Medical Center, Tainan, TWN; 3 Nutrition, Department of Nutrition, Chi Mei Medical Center, Tainan, TWN

**Keywords:** covid-19, intensive-care unit, mortality, sars-cov-2, vitamin-d deficiency

## Abstract

Introduction: Vitamin D deficiency (VDD) is prevalent worldwide and may influence the severity of infectious diseases, including COVID-19. This study aimed to evaluate the association between VDD and 30-day clinical outcomes in patients with COVID-19.

Methods: We conducted a retrospective cohort study using data from the TriNetX database, which includes de-identified electronic health records of approximately 155 million patients from 131 healthcare organizations globally. Adult patients (aged ≥18 years) with their first documented COVID-19 infection between January 2022 and September 2024 were included. Patients were categorized based on their vitamin D status within three months prior to COVID-19 diagnosis: those with documented VDD (VDD group) and those without (controls). Outcomes assessed were all-cause mortality, all-cause hospitalization, the requirement for critical care services, and a composite outcome of these events at 30-day follow-up. Propensity score matching (PSM) was utilized to balance covariates such as age, sex, race, and comorbidities between the groups. Hazard ratios (HRs) with 95% confidence intervals (CIs) were calculated using Cox regression analysis.

Results: After PSM, the study included 68,814 patients, with 34,407 in both the VDD and control groups. VDD was associated with a slight but statistically significant increase in the hazard of experiencing the composite outcome (HR 1.05, 95% CI 1.01-1.10, P = 0.02). There was no significant difference in all-cause mortality (HR 1.06, 95% CI 0.92-1.22, P = 0.42) or all-cause hospitalization (HR 1.01, 95% CI 0.96-1.05, P = 0.80) between the groups. However, patients with VDD had a significantly higher hazard of requiring critical care services compared to controls (HR 1.28, 95% CI 1.16-1.41, P < 0.01).

Conclusion: Vitamin D deficiency is associated with an increased risk of requiring critical care services among COVID-19 patients, suggesting that VDD may contribute to greater disease severity. These findings underscore the potential importance of assessing and managing vitamin D deficiency in patients with COVID-19 to improve clinical outcomes.

## Introduction

Coronavirus disease 2019 (COVID-19), caused by the severe acute respiratory syndrome coronavirus 2 (SARS-CoV-2), emerged in late 2019 and swiftly escalated into a global pandemic [1]. COVID-19 continues to pose significant challenges to global health, with millions of confirmed cases and substantial mortality worldwide. The disease spectrum ranges from asymptomatic infection to severe respiratory failure and death. Identifying factors that influence disease severity and patient outcomes is crucial for improving clinical management and reducing mortality [2].

Vitamin D, a fat-soluble secosteroid, is essential for calcium homeostasis and bone metabolism. Beyond its classical roles, vitamin D has been recognized for its immunomodulatory properties. It influences both innate and adaptive immune responses by regulating the production of antimicrobial peptides and modulating the activity of immune cells such as macrophages, dendritic cells, and T lymphocytes. Vitamin D receptors are widely expressed on immune cells, underscoring its potential impact on immune function. Vitamin D deficiency (VDD) is a widespread public health issue, affecting approximately one billion people globally. Risk factors include limited sun exposure, higher latitudes, darker skin pigmentation, aging, obesity, and certain chronic diseases. The prevalence of VDD raises concerns about its potential impact on infectious diseases, particularly respiratory infections [3].

Several studies have suggested a link between low vitamin D levels and increased susceptibility to acute respiratory tract infections [4]. Vitamin D supplementation has been shown to reduce the risk of respiratory infections by enhancing mucosal defenses and modulating inflammatory responses [4]. In the context of COVID-19, emerging evidence indicates that VDD may be associated with worse clinical outcomes [3]. Observational studies have reported higher rates of severe disease, intensive care unit (ICU) admission, and mortality among COVID-19 patients with low vitamin D levels [5].

Despite these findings, the relationship between VDD and COVID-19 outcomes remains inconclusive due to methodological limitations in existing studies, such as small sample sizes, confounding factors, and variability in defining VDD. Randomized controlled trials are limited, and there is a need for more robust evidence to establish their association [3-5].

This study aims to address these gaps by utilizing a large, multi-institutional database (TriNetX, Cambridge, MA, USA) to evaluate short-term clinical outcomes in COVID-19 patients with and without VDD. By leveraging a comprehensive dataset and applying rigorous statistical methods, our study seeks to provide clearer evidence on the impact of VDD on critical outcomes such as all-cause mortality, hospitalization, and the need for critical care services. Understanding the short-term effects of VDD in COVID-19 patients is essential to inform clinical management and potential preventive strategies, including vitamin D supplementation.

## Materials and methods

Database source

We conducted a retrospective cohort study using data from the TriNetX multi-institutional database, a global federated research network that provides access to de-identified electronic health records (EHRs) of approximately 155 million patients across 131 healthcare organizations worldwide [6]. Clinical data were extracted through an integrated natural language processing system applied to EHRs and clinical documents. To ensure patient confidentiality, the TriNetX platform supplies only aggregate counts and statistical summaries, maintaining de-identification throughout the research process. The study was approved by the Institutional Review Board of the Chi Mei Medical Center (approval no. 11302-E01).

Study design/inclusion and exclusion criteria

Adult patients who suffered from delta-COVID-19 were identified on the TriNetX Diamond Network from January 2022 to September 2024. We identified adult patients aged 18 years and older who experienced their first documented COVID-19 infection during this period, which was designated as the index date [7]. Patients were categorized into two cohorts based on their vitamin D status within the three months preceding their COVID-19 diagnosis: those with documented VDD (25-hydroxyvitamin D cutoff < 20 ng/mL) and those without [8]. Patients lacking documentation of their vitamin D status were excluded from the analysis.

Outcomes

The primary objective of the study was to evaluate one-month outcomes in COVID-19 patients with VDD compared to those without VDD. The outcomes assessed included all-cause mortality, all-cause hospitalization, the requirement for critical care services (based on CPT codes: 1013729, 1014309, or 99291), and a composite outcome encompassing these events at 30-day follow-up after COVID-19 infection.

Statistical analysis

Statistical analyses were conducted using TriNetX Live, a browser-based, real-time analytics platform. Baseline characteristics were summarized using means and standard deviations for continuous variables and frequencies and percentages for categorical variables. Covariates considered for analysis included demographic factors (age, sex, race) and comorbid conditions. To control for potential confounding variables and balance the cohorts, we employed one-to-one propensity score matching (PSM) using a nearest-neighbor matching algorithm without replacement. Hazard ratios (HRs) with 95% confidence intervals (CIs) were calculated for each outcome using Cox regression [9].

## Results

The study initially included 145,372 patients diagnosed with COVID-19, comprising 110,969 individuals with VDD and 34,403 control patients without VDD (Table 1). Before PSM, the mean age was similar between the VDD and control groups (58.2 ± 17.7 years vs. 58.3 ± 18.1 years; P = 0.41). However, significant differences were observed in sex distribution and comorbidities. The control group had a higher proportion of females compared to the VDD group, with 23,551 (68.5%) females in the control group versus 68,815 (65.5%) in the VDD group (P < 0.01). Conversely, the VDD group had a slightly higher proportion of males, 31,226 (29.7%) compared to 10,535 (30.6%) in the control group (P < 0.01).

**Table 1:** Baseline characteristics of the study population before and after propensity score matching Continuous variables were compared using an independent t-test, and categorical variables were compared using a chi-square test. VDD: vitamin D deficiency

Variables	Before matching			After matching		
	VDD group (n=110,969)	Control group (n=34,407)	P value	VDD group (n=34,407)	Control group (n=34,407)	P value
Age at index, years						
Mean ± SD	58.2 ± 17.7	58.3 ± 18.1	0.41	58.2 ± 18.1	58.3 ± 18.1	0.75
Sex, n(%)						
Female	68,815 (65.5)	23,551 (68.5)	< 0.01	23,673 (68.8)	23,550 (68.5)	0.31
Male	31,226 (29.7)	10,535 (30.6)	< 0.01	10,410 (30.3)	10,535 (30.6)	0.30
Race, n(%)						
White	64,454 (61.3)	23,170 (67.3)	< 0.01	23,416 (68.1)	23,170 (67.3)	0.04
Black or African American	16,774 (16)	2,899 (8.4)	< 0.01	2,811 (8.2)	2,899 (8.4)	0.02
Asian	3,956 (3.8)	1,558 (4.5)	< 0.01	1,351 (3.9)	1,558 (4.5)	< 0.01
Comorbidities, n(%)						
Hypertensive diseases	55,078 (52.4)	15,302 (44.5)	< 0.01	15,139 (44)	15,302 (44.5)	0.21
Essential (primary) hypertension	51,940 (49.4)	14,170 (41.2)	< 0.01	14,264 (41.5)	14,170 (41.2)	0.47
Overweight and obesity	28,630 (27.2)	6,629 (19.3)	< 0.01	6,535 (19)	6,629 (19.3)	0.36
Chronic lower respiratory diseases	22,119 (21)	5,855 (17)	< 0.01	5,828 (16.9)	5,855 (17)	0.78
Ischemic heart diseases	15,254 (14.5)	4,786 (13.9)	< 0.01	4,601 (13.4)	4,785 (13.9)	0.04
Heart failure	9,768 (9.3)	2,940 (8.5)	< 0.01	2,676 (7.8)	2,940 (8.5)	< 0.01
Cerebrovascular diseases	7,914 (7.5)	2,279 (6.6)	< 0.01	2,062 (6)	2,279 (6.6)	< 0.01
End stage renal disease	2,952 (2.8)	1,211 (3.5)	< 0.01	1,034 (3)	1,210 (3.5)	< 0.01

Racial composition also differed significantly. The control group had a higher percentage of White patients, 23,170 (67.3%) compared to 64,454 (61.3%) in the VDD group (P < 0.01). The VDD group had more Black or African American patients, 16,774 (16%) versus 2,899 (8.4%) in the control group (P < 0.01), and a slightly lower percentage of Asian patients, 3,956 (3.8%) compared to 1,558 (4.5%) (P < 0.01).

Comorbidities were more prevalent in the VDD group before matching. Hypertensive diseases were present in 55,078 (52.4%) patients in the VDD group compared to 15,302 (44.5%) in the control group (P < 0.01). Essential hypertension was higher in the VDD group, with 51,940 (49.4%) patients versus 14,170 (41.2%) in the control group (P < 0.01). Overweight and obesity were more common among VDD patients, 28,630 (27.2%) compared to 6,629 (19.3%); P < 0.01. Chronic lower respiratory diseases were more prevalent in the VDD group, with 22,119 (21%) patients versus 5,855 (17%) in the control group (P < 0.01). Similarly, ischemic heart diseases were reported in 15,254 (14.5%) patients in the VDD group compared to 4,786 (13.9%) in the control group (P < 0.01). Heart failure was present in 9,768 (9.3%) patients in the VDD group versus 2,940 (8.5%) in the control group (P < 0.01), and cerebrovascular diseases were noted in 7,914 (7.5%) patients in the VDD group compared to 2,279 (6.6%) in the control group (P < 0.01). Interestingly, end-stage renal disease was slightly less prevalent in the VDD group, with 2,952 (2.8%) patients compared to 1,211 (3.5%) in the control group (P < 0.01).

After PSM, the cohorts were balanced with 34,407 patients in each group. The mean age remained similar between the VDD and control groups (58.2 ± 18.1 years vs. 58.3 ± 18.1 years; P = 0.75). Sex distribution was nearly identical post-matching, with females comprising 23,673 (68.8%) of the VDD group and 23,550 (68.5%) of the control group (P = 0.31), and males comprising 10,410 (30.3%) and 10,535 (30.6%), respectively (P = 0.30).

Racial composition was also balanced after matching. White patients represented 23,416 (68.1%) of the VDD group and 23,170 (67.3%) of the control group (P = 0.04). Black or African American patients accounted for 2,811 (8.2%) in the VDD group and 2,899 (8.4%) in the control group (P = 0.02). Asian patients comprised 1,351 (3.9%) of the VDD group and 1,558 (4.5%) of the control group (P < 0.01).

The prevalence of comorbidities was largely comparable between the matched groups. Hypertensive diseases were present in 15,139 (44%) patients in the VDD group and 15,302 (44.5%) in the control group (P = 0.21). Essential hypertension was reported in 14,264 (41.5%) patients in the VDD group versus 14,170 (41.2%) in the control group (P = 0.47). Overweight and obesity were similar between the groups, with 6,535 (19%) patients in the VDD group and 6,629 (19.3%) in the control group (P = 0.36). Chronic lower respiratory diseases were present in 5,828 (16.9%) patients in the VDD group and 5,855 (17%) in the control group (P = 0.78). Slight differences were noted in ischemic heart diseases, with 4,601 (13.4%) patients in the VDD group compared to 4,785 (13.9%) in the control group (P = 0.04). Heart failure was observed in 2,676 (7.8%) patients in the VDD group versus 2,940 (8.5%) in the control group (P < 0.01). Cerebrovascular diseases were reported in 2,062 (6%) patients in the VDD group compared to 2,279 (6.6%) in the control group (P < 0.01), and end-stage renal disease was present in 1,034 (3%) patients in the VDD group versus 1,210 (3.5%) in the control group (P < 0.01).

After PSM, we assessed the association between VDD and clinical outcomes in COVID-19 patients (Table 2). The primary composite outcome occurred in 4,345 patients (12.6%) in the VDD group and 4,181 patients (12.1%) in the control group. VDD was associated with a slight but statistically significant increase in the hazard of experiencing the composite outcome (HR 1.05, 95% CI 1.01-1.10, P = 0.02).

**Table 2:** Hazard ratios of clinical outcomes for the matched vitamin D deficiency group and the control group A Cox regression model was used to compare the hazard ratio between the two groups. An alpha level of 0.05 was selected for statistical significance. CI: confidence interval; VDD: vitamin D deficiency

Outcomes	Patients with outcome (n)		Hazard ratio (95% CI)	P value
	VDD group	Control group		
Primary outcome				
Composite outcome	4,345	4,181	1.05 (1.01 to 1.10)	0.02
Secondary outcomes				
All-cause mortality	382	365	1.06 (0.92 to 1.22)	0.42
All-cause hospitalization	3,803	3,812	1.01 (0.96 to 1.05)	0.80
Requiring critical care services	885	702	1.28 (1.16 to 1.41)	< 0.01

**Figure 1:**
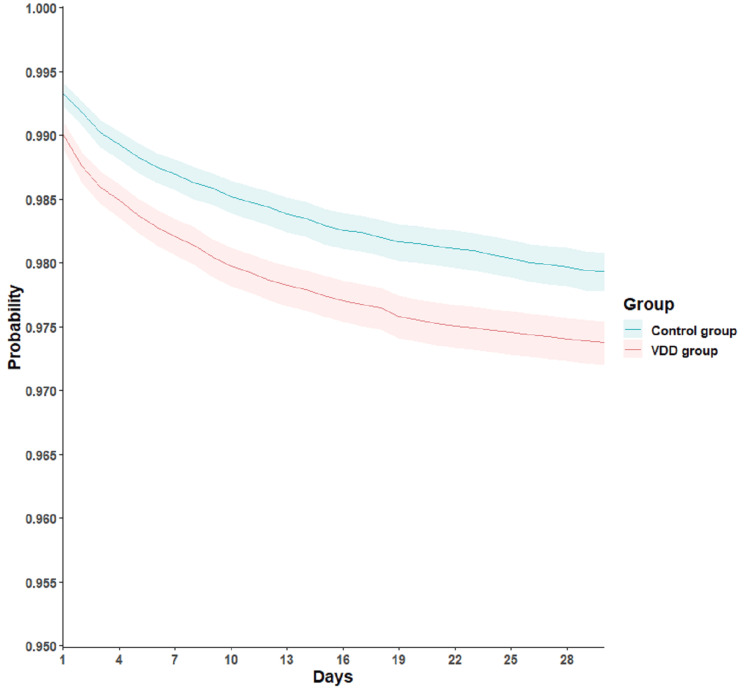
Kaplan–Meier time-to-event free curves for the requiring critical care services VDD: vitamin D deficiency

For the secondary outcomes, there was no significant difference in incidence of all-cause mortality between the VDD group (382 patients, 1.1%), and the control group (365 patients, 1.1%), yielding an HR of 1.06 (95% CI 0.92-1.22, P = 0.42). Similarly, all-cause hospitalization rates were comparable between the VDD group, with 3,803 patients (11.1%), and the control group, with 3,812 patients (11.1%), resulting in an HR of 1.01 (95% CI 0.96-1.05, P = 0.80). However, a notable finding was that patients with VDD had a significantly higher hazard of requiring critical care services compared to those without deficiency. Specifically, 885 patients (2.6%) in the VDD group required critical care versus 702 patients (2%) in the control group, corresponding to an HR of 1.28 (95% CI 1.16-1.41, P < 0.01; Table 2 and Figure 1).

## Discussion

In this large retrospective cohort study utilizing a propensity score-matched population, we found that VDD in COVID-19 patients was associated with a slight but statistically significant increase in the risk of adverse short-term outcomes. Specifically, VDD was linked to a higher hazard of experiencing the composite outcome of all-cause mortality, hospitalization, and the requirement for critical care services (HR 1.05, 95% CI 1.01-1.10, P = 0.02). Notably, while there was no significant difference in all-cause mortality or hospitalization rates between the VDD and control groups, patients with VDD had a significantly higher hazard of requiring critical care services (HR 1.28, 95% CI 1.16-1.41, P < 0.01).

These findings suggest that VDD may contribute to the severity of COVID-19 illness, necessitating more intensive medical interventions such as critical care. The lack of significant differences in all-cause mortality and hospitalization rates may indicate that while VDD influences disease severity, it does not necessarily affect the overall survival or the likelihood of hospitalization in the short term. However, the increased need for critical care services highlights the potential role of vitamin D in modulating the clinical course of COVID-19.

While VDD may exacerbate the severity of COVID-19, leading to critical events that necessitate intensive intervention (e.g., respiratory support), these interventions may still mitigate mortality in the short term. Furthermore, hospitalization rates may not differ significantly if patients with and without VDD present similarly in the early stages of infection, but those with VDD subsequently progress to more severe conditions requiring intensive care.

Our results align with several observational studies that have reported associations between low vitamin D levels and increased severity of COVID-19 [10,11]. For instance, a study by Merzon et al. found that low plasma 25(OH)D levels were independently associated with the likelihood of COVID-19 infection and hospitalization [12]. Another study by Kiyumi et al. reported that VDD could be a risk factor for increased susceptibility to COVID-19 infection and severity [13]. These studies, along with our findings, support the hypothesis that adequate vitamin D levels may play a protective role against severe manifestations of COVID-19.

The proposed mechanisms by which vitamin D may influence COVID-19 outcomes include the suppression of the renin-angiotensin system, reduction of pro-inflammatory cytokine production, and mitigation of the cytokine storm-a hyperinflammatory state contributing to severe disease manifestations. Vitamin D may also enhance cellular immunity and promote the expression of genes involved in antioxidant pathways, potentially reducing viral replication and tissue damage [5].

The mechanisms by which vitamin D may influence COVID-19 outcomes are multifaceted. Vitamin D is known to modulate the immune system by enhancing innate immunity and regulating adaptive immunity. It promotes the production of antimicrobial peptides such as cathelicidin and defensins, which can lower viral replication rates [14]. Additionally, vitamin D suppresses the production of pro-inflammatory cytokines like interleukin-6 and tumor necrosis factor-alpha, potentially mitigating the cytokine storm associated with severe COVID-19 cases [15]. Vitamin D also influences the renin-angiotensin system by downregulating the expression of renin, which may have implications in COVID-19 pathophysiology given the virus's interaction with the ACE2 receptor [14-16].

Despite the strengths of our study, including a large sample size and the use of propensity score matching to balance confounding variables. The findings of this study have important clinical and public health implications. Given the relatively high prevalence of VDD worldwide and its potential association with severe COVID-19 outcomes, screening for and correcting VDD in at-risk populations may be a cost-effective strategy to improve patient outcomes. However, randomized controlled trials are necessary to determine whether vitamin D supplementation can reduce the severity of COVID-19 and to establish appropriate dosing guidelines.

However, there are limitations that should be acknowledged. First, the retrospective design inherently carries the risk of residual confounding and cannot establish causality. Second, vitamin D status was determined based on documentation within three months prior to COVID-19 diagnosis, which may not reflect the vitamin D levels at the time of infection. Third, the study relies on electronic health records, which may have inconsistencies or missing data. Additionally, the definition of VDD and the lack of standardized measurements across different healthcare organizations may introduce variability. Another limitation is that we did not account for vitamin D supplementation during the course of the illness, which could have affected outcomes. Furthermore, while we adjusted for several comorbidities, unmeasured confounders such as socioeconomic status, nutritional status, and other lifestyle factors could influence both vitamin D levels and COVID-19 outcomes. Additionally, the variability in vitamin D levels across different geographic and ethnic groups could influence the association between VDD and COVID-19 severity. Vitamin D levels are affected by factors such as sun exposure, latitude, skin pigmentation, dietary habits, and genetic predispositions. These differences may lead to variability in VDD prevalence and its impact on COVID-19 outcomes across populations. For example, populations residing in higher latitudes with limited sun exposure or those with darker skin pigmentation are more prone to VDD. These factors could contribute to differences in disease severity, and as such, future studies should consider stratifying analyses based on geographic and ethnic variations to provide a more nuanced understanding of the relationship between VDD and COVID-19.

## Conclusions

In this large propensity score-matched retrospective cohort study, we found that VDD is significantly associated with an increased risk of requiring critical care services among patients with COVID-19. Specifically, patients with VDD had a 28% higher hazard of needing critical care compared to those without deficiency. While there were no significant differences in all-cause mortality or all-cause hospitalization rates between the groups, the elevated need for critical care underscores the impact of vitamin D status on disease severity.

These findings highlight VDD as a potential modifiable risk factor in the management of COVID-19. Assessing and correcting VDD in patients may reduce disease severity, decrease the demand for critical care resources, and improve clinical outcomes. Given the widespread prevalence of VDD globally, incorporating vitamin D status evaluation into routine clinical assessments for COVID-19 patients could have significant public health implications.

Our study emphasizes the need for heightened awareness of VDD in the context of COVID-19 and supports further research to explore the benefits of vitamin D supplementation as a therapeutic strategy to mitigate severe outcomes in affected patients.

To confirm the potential benefits of vitamin D supplementation and establish appropriate dosing strategies, randomized controlled trials (RCTs) are essential. Future research should focus on determining the optimal vitamin D dosage, timing of supplementation, and its effects on different patient subgroups, particularly those at higher risk for severe COVID-19 outcomes. These studies could provide the evidence needed to guide clinical practice and improve patient management during the ongoing pandemic.
